# Unveiling the potential of Butylphthalide: inhibiting osteoclastogenesis and preventing bone loss

**DOI:** 10.3389/fphar.2024.1347241

**Published:** 2024-02-23

**Authors:** Feng Yanbin, Teng Yilin, Mo Yaomin, Xi Deshuang, Zhou Junhong, Zeng Gaofeng, Zong Shaohui

**Affiliations:** ^1^ Department of Spine Osteopathia, The First Affiliated Hospital of Guangxi Medical University, Guangxi Medical University, Nanning, Guangxi, China; ^2^ Department of Spine, Guangxi Medical University, Nanning, Guangxi, China; ^3^ Department of Nutrition and Food Hygiene, College of Public Hygiene of Guangxi Medical University, Nanning, Guangxi, China

**Keywords:** Butylphthalide, osteoclast, ROS, osteoblast, osteoporosis

## Abstract

Osteoporosis, resulting from overactive osteoclasts and leading to elevated fracture risk, has emerged as a global public health concern due to the aging population. Therefore, inhibiting osteoclastogenesis and bone resorption function represents a crucial approach for preventing and treating osteoporosis. The purpose of this study was to examine the effects and molecular mechanisms of Butylphthalide (NBP) on the differentiation and function of osteoclasts induced by RANKL. Osteoclastogenesis was assessed through TRAP staining and bone slice assay. An animal model that underwent ovariectomy, simulating postmenopausal women’s physiological characteristics, was established to investigate the impact of Butylphthalide on ovariectomy-induced bone loss. To delve deeper into the specific mechanisms, we employed Western blot, PCR, immunofluorescence, and immunohistochemical staining to detect the expression of proteins that are associated with the osteoclast signaling pathway. In this study, we found that Butylphthalide not only suppressed osteoclastogenesis and bone resorption *in vitro* but also significantly decreased TRAcP-positive osteoclasts and prevented bone loss *in vivo*. Further mechanistic experiments revealed that Butylphthalide reduces intracellular ROS in osteoclasts, inhibits the MAPK and NFATc1 signaling pathways, and downregulates the key genes and proteins of osteoclasts. This inhibits osteoclast formation and function. The reduction in ROS in osteoclasts is intricately linked to the activity of Butylphthalide-modulated antioxidant enzymes. Overall, NBP may offer a alternative treatment option with fewer side effects for skeletal diseases such as osteoporosis.

## 1 Introduction

Osteoporosis is characterized by a decrease in the density of the bone and a deterioration in the microstructure of the bone, leading to skeletal fragility and increasing the risk of fractures (Johnston and Dagar, 2020; Zhang et al., 2022a). This could pose a significant health risk to elderly individuals, especially considering the high prevalence of osteoporosis in this demographic. Osteoporosis results from a disruption in the equilibrium between bone formation and resorption (Li H. et al., 2021). Osteoclasts affect bone resorption, and excessive resorption leads to microstructural deterioration (Shih and Varghese, 2019). Osteoclasts are derived from hematopoietic stem cells under the action of M-CSF and RANKL (Li B. et al., 2022; Feng et al., 2022; Kawai et al., 2023). M-CSF promotes precursor cell survival and proliferation, while RANKL regulates osteoclast differentiation (Feng et al., 2019; Mun et al., 2020; Ono et al., 2020). RANKL binds to surface receptors and activates osteoclast-related signaling cascades, inducing generation and differentiation (Zhou et al., 2019; Montaseri et al., 2020; Elango et al., 2021). Therefore, inhibiting RANKL downstream signaling might be an effective treatment strategy for osteoporosis.

Intracellular reactive oxygen species (ROS) play a vital role in regulating cellular operations. They serve as significant second messengers that modulate signaling pathways, cell differentiation, and apoptosis (Liu et al., 2019; Ma et al., 2019). Additionally, ROS play a pivotal role in influencing osteoclastogenesis. RANKL binds to specific receptors and influences the activation of the TRAF6 and Nox1 signaling pathways, thereby upregulating ROS levels alongside Rac1. ROS affect osteoclasts through downstream PI3K, NFKB, and MAPK pathways. Thus, decreasing the elevation of ROS levels mediated by RANKL can effectively inhibit osteoclast formation. When intracellular levels of ROS are increased, the Keap1-Nrf2 pathway reduces ROS via the increased expression of antioxidant enzymes (Cheon et al., 2020; Weivoda and Bradley, 2023). This suggests that antioxidant therapy can suppress osteoclastogenesis and prevent bone loss (Jeong et al., 2019; Iantomasi et al., 2023).

Nevertheless, regenerating damaged bone tissue in osteoporosis patients remains a therapeutic challenge. Conventional anti-osteoporotic medications can improve conditions to some degree but cannot effectively repair affected areas, with long-term use incurring more side effects (Li J. et al., 2022; Chen et al., 2022; Long et al., 2022; Wu et al., 2022). Therefore, searching for a safe and efficacious osteoporosis drug is imperative. Butylphthalide (NBP, 3-n-Butylphthalide) is a compound initially isolated from the seeds of the celery (*Apium graveolens* Linn, from the Umbelliferae family) (Abdoulaye and Guo, 2016; Chen X. Q. et al., 2019). Later, researchers found NBP in the traditional Chinese medicine Angelica sinensis, which also belongs to the Umbelliferae family, however, the content was not as high as that of celery seeds (Wei et al., 2016). Since its discovery, NBP has garnered significant interest from the scientific community for its therapeutic role in cerebrovascular diseases, including increasing cerebral blood flow and protecting neuronal cells (Tan et al., 2022; Guo et al., 2023; Lyu et al., 2023). It also reduces inflammation following brain injury (Jia J. et al., 2023). NBP inhibits oxidative stress by activating the Nrf2/HO-1 pathway and suppressing the MAPK pathway, thereby reducing ischemia-induced brain damage (Li et al., 2023; Min et al., 2023). Additionally, NBP exhibits neuroprotective properties by reducing ROS in nerve cells after a stroke (Jia Y. et al., 2023). As a result, the Chinese Food and Drug Administration has authorized the use of NBP for stroke treatment. However, no studies have yet established a relationship between NBP and osteoclasts. Based on the reviewed literature, it is hypothesized that NBP inhibits the differentiation and formation of osteoclasts by inhibiting the ROS and MAPK pathways. This ultimately prevents the excess of osteoclasts and osteoporosis.

## 2 Materials and methods

### 2.1 Reagents and media

Butylphthalide (NBP; HY-B0647; CAS No. 6066-49-5; 99.98% purity) was purchased from Med Chem Express. The Certificates of Analytical, Safety Data Sheet, HNMR and LCMS of Butylphthalide are shown in Supplementary Figures. NBP was dissolved to 10 mM using dimethyl sulfoxide and stored at −80°C. α-MEM medium for cell culture was supplied by Thermo Fisher Scientific (Massachusetts, United States), fetal bovine serum (FBS) was supplied by Avantor, and R&D Systems provided RANKL and M-CSF. PBS and 5% bovine serum albumin solution were provided by Solarbio. Cell Signaling Technology provided antibodies targeting p-ERK, ERK, P38, p-P38, p-JNK, and JNK. NFATc1, c-Fos, CTSK, p-P65, P65, and IκB-α antibodies were provided by Abcam; HO-1, Nrf2, GSR, and CAT were supplied by Beyotime Biotechnology. β-Actin was provided by Sevicebio. Fluorescent secondary antibodies were supplied by Thermo Fisher Scientific.

### 2.2 Cell extraction and osteoclastogenesis

Bone marrow stromal cells (BMMs) were obtained from the lower limb bones of 6-week-old mice from the Experimental Animal Center. Medium supplemented with 1% P-S, 10% FBS, and 30 ng/ml M-CSF was used to cultivate BMMs for 4 days (5% CO2, 37°C). After 2 days, the medium was replaced once. In 96-well plates, adherent cells were collected and inoculated 6 days later at 7 × 10^3^ cells per well. The culture medium with RANKL was exchanged every 2 days, and NBP of variable concentrations was added to investigate the drug effects on osteoclast differentiation. Fixed cells were treated with a 4% paraformaldehyde solution for 30 min and subsequently stained using TRAcP reagent. Images captured by the EVOS FL Auto2 imaging system (Thermo Fisher Scientific, United States) were analyzed using ImageJ v1.8.0.

### 2.3 Cytotoxicity assay

BMMs were seeded in 96-well plates and cultivated in medium containing graded NBP concentrations (0, 5, 10, 15, 20, and 25 μM) for 48 and 96 h. After adding 10 μL CCK-8 reagent to each well, the plate was incubated for an hour. The absorbance at 450 nm was then measured using a microplate reader.

### 2.4 Podosomal actin belt immunofluorescence and intracellular ROS detection

BMMS inoculated in 96-well plates were cultured for 48 h using media containing different concentrations of NBP (0, 5, 10, and 20 μM). The Reactive Oxygen Kit was utilized to measure intracellular ROS levels (Biosharp, China). An EVOS imaging system was used to acquire the images, and the intracellular ROS fluorescence intensity was quantified with ImageJ v1.8.0. For actin ring staining, 4% paraformaldehyde solution was used to fix the cells for 30 min, followed by 0.1% Triton X-100 treatment for 5 min. BSA blocking (3%) was added for 1 hour, followed by rhodamine-conjugated phalloidin incubation for 2 h. Finally, the cells were rinsed with 0.2% BSA and stained with DAPI for 5 min in darkness. The samples were then observed using the EVOS FL Auto2 imaging system, quantifying the osteoclast number and area.

### 2.5 Bone pit resorption assay

Bone slices were placed in 96-well plates and inoculated at 1 × 10^4^ cells/well in complete medium (30 ng/mL M-CSF, 50 ng/mL RANKL) with variable NBP concentrations (0, 5, 10, and 20 μM) for 5 days. An electron microscope was used to visualize the samples (Hitachi Ltd. Regulus 8100), and the absorbed area of the bovine bone slices was calculated by ImageJ v1.8.0.

### 2.6 RNA extraction and analysis

After 6 days of NBP intervention, the cells underwent lysis through the addition of TRIzol reagent. Total RNA was subsequently extracted. Total RNA was reverse transcribed to cDNA using the cDNA Synthesis Kit (Thermo Fisher Scientific, United States). The amplification of specific sequences was performed using the StepOne System (Thermo Fisher Scientific, United States) following the manufacturer’s protocol. The primers used are listed in Table 1.

**TABLE 1:** The specific primers used in qRT-PCR.

Gene	NCBI ID	Primer Sequence (5-3′)	
		Forward	Reverse
Nfatc1	18,018	GGT​GCT​GTC​TGG​CCA​TAA​CT	GAA​ACG​CTG​GTA​CTG​GCT​TC
Fos	14,281	CCA​GTC​AAG​AGC​ATC​AGC​AA	AAG​TAG​TGC​AGC​CCG​GAG​TA
Ctsk	13,038	AGG​CGG​CTC​TAT​ATG​ACC​ACT​G	TCT​TCA​GGG​CTT​TCT​CGT​TC
Mmp9	17,395	CGT​GTC​TGG​AGA​TTC​GAC​TTG​A	TTG​GAA​ACT​CAC​ACG​CCA​GA
Atp6v0d2	242,341	GTC​CCA​TTC​TTG​AGT​TTG​AGG	GGA​TAG​AGT​TTG​CCG​AAG​GTT
Acp5	11,433	TGTGGCCATCTTTATGCT	GTCATTTCTTTGGGGCTT
GAPDH	14,433	GGC​ACA​GTC​AAG​GCT​GAG​AAT​G	ATG​GTG​GTG​AAG​ACG​CCA​GTA

### 2.7 Protein extraction and western blotting

Cells were lysed with the predetermined cell lysis solution (1% PMSF, 1% phosphatase inhibitor and 1% protease inhibitor) at 4°C for 5 min, centrifuged using the Protein Extraction Kit-Column Method at 15,000 × g for 30 s at 4°C, and blended with loading buffer, followed by heating at 95°C for 10 min. Electrophoresis (120 V, 30 min) was then conducted on a 10% precast polyacrylamide gel. The proteins from the gel were subsequently transferred onto a PVDF membrane. (Merck, Germany) (220 V, 120 min), which was then blocked with 5% skim milk for 1.5 h. Samples, along with specific primary antibodies, were incubated on a shaker at 4°C for 12 h and then incubated for 1 h with fluorescence-labeled secondary antibodies. Final scans were performed using an Odyssey (LI-COR Biosciences, United States) near-infrared fluorescence imaging scanner, and the band gray values were measured by ImageJ v1.8.0.

### 2.8 Fluorescence staining for NFATc1 nuclear translocation

BMMs were inoculated in culture dishes with medium containing M-CSF, RANKL, and variable NBP concentrations for 7 days. A 4% paraformaldehyde solution was utilized to preserve the cells for 30 min, and then 0.1% Triton X-100% and 3% BSA were added and incubated for 5 min and 20 min, respectively. The samples were washed in 0.2% BSA solution and then incubated overnight with NFATc1 antibodies. On the following day, fluorescent secondary antibodies were introduced for incubation and DAPI staining and visualized with the EVOS FL Auto2 imaging system.

### 2.9 OVX-induced osteoporosis animal model

Thirty 10-week-old female C57BL/6 mice were selected and divided into the sham surgery, vehicle, E2, low-dose drug (10 mg/kg), and high-dose drug (20 mg/kg) groups, including 6 mice per group. Mice were anesthetized with 2% tribromoethanol before bilateral dorsal incisions were made for ovariectomy except for the sham group. The E2 group was administered 50 ng/kg E2 intraperitoneally every other day starting 7 days postoperation. The drug groups received NBP treatment, and the sham and vehicle groups received saline. After 6 weeks of treatment, all mouse femurs were collected for analysis. Guangxi Medical University’s Animal Ethics Committee reviewed and approved all animal experimentation protocols (No. 202209116).

### 2.10 Micro-CT scanning and analysis

The excised mouse femurs were analyzed by a CT-100 scanner with 65 kV voltage and 9 μm pixels. The 3D femur images were reconstructed by Skyscan CT software (Bruker, Belgium), and the growth plate subchondral area was delineated for bone parameter analysis.

### 2.11 Pathohistological staining analysis

Femoral samples were embedded 2 days after decalcification and then cut into 5 μm paraffin sections. Sections were then processed using HE, TRAcP and immunohistochemical staining (HO-1, Nrf2) and analyzed by ImageJ v1.8.0, measuring parameters such as positive area/bone tissue, bone area/tissue area (BS/TS) and osteoclast number/bone trabecular area (N.Oc/BS).

### 2.13 Statistical analysis

Each experiment was performed a minimum of three times. Data are presented as the mean ± SD. GraphPad Prism 8.0 was utilized for statistical analysis using ANOVA or Student’s t test. *p* < 0.05 was considered statistically significant.

## 3 Result

### 3.1 NBP inhibits RANKL-induced OC differentiation *in vitro*

**FIGURE 1:**
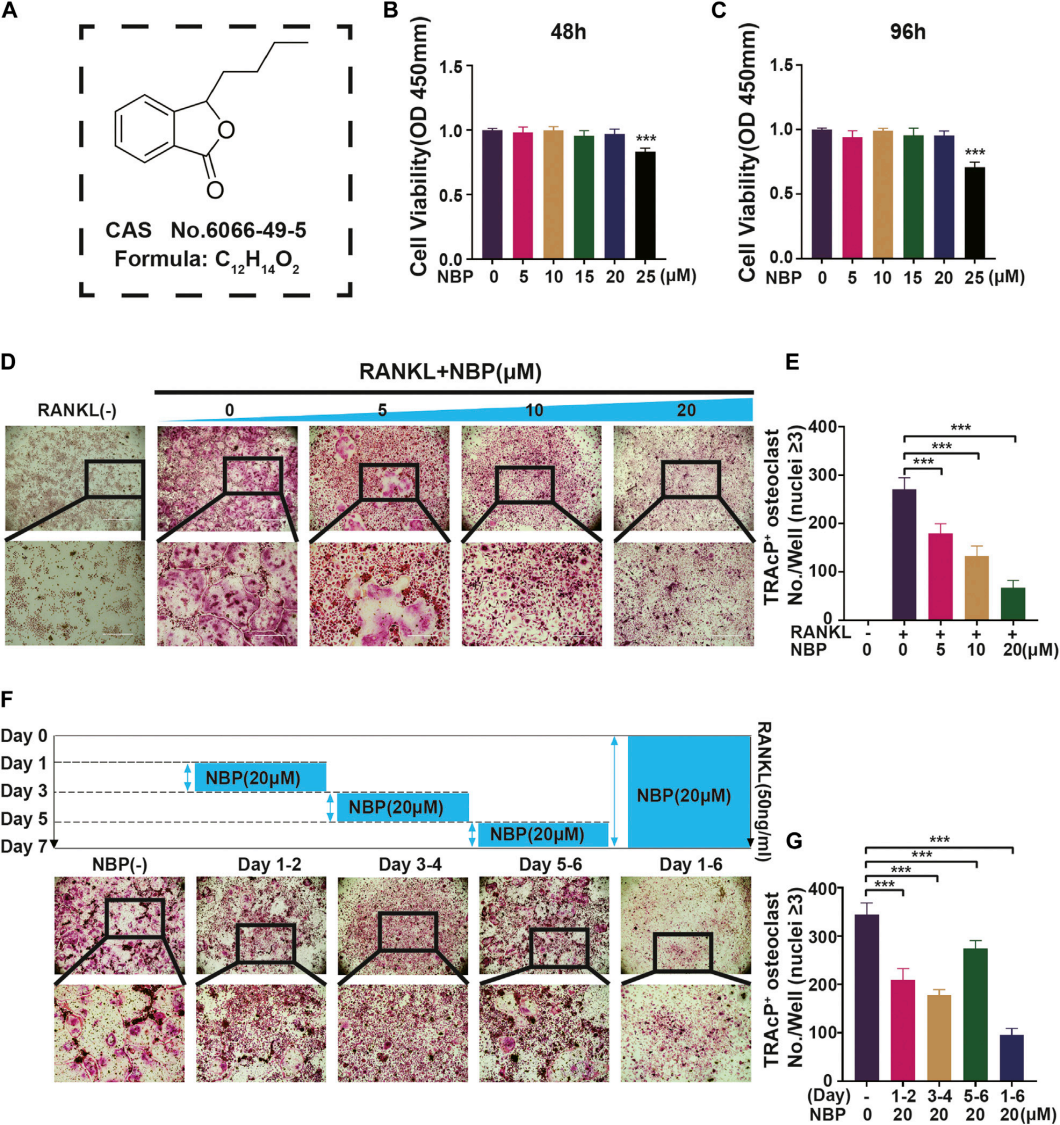
NBP repress RANKL-induced osteoclastogenesis *in vitro*. **(A)** An illustration of NBP’s molecular structure. **(B,C)** Cell activity was determined by the CCK-8 assay following exposure to various concentrations of NBP on BMMS for 48 and 96 h. **(D)** Representative images of TRAcP demonstrated that NBP suppressed the osteoclastogenesis within 7 days of RANKL (50 ng/mL) stimulation (scale bar = 1000 µm). **(E)** The number of cells with positive TRAcP markers was quantitatively analyzed (nuclei ≥ 3). **(F)** Representative images of TRAcP revealed that NBP (20 µM) inhibited the process of osteoclastogenesis during specific time periods (scale bar = 1000 µm). **(G)** TRAcP^+^ cells in each well at different time points (nuclei ≥ 3). *p < 0.05, **p < 0.01, ***p < 0.001 relative to the 0 µM NBP. All data of the bar are presented as the mean ± SD (*n* = 3 per group).

**FIGURE 2:**
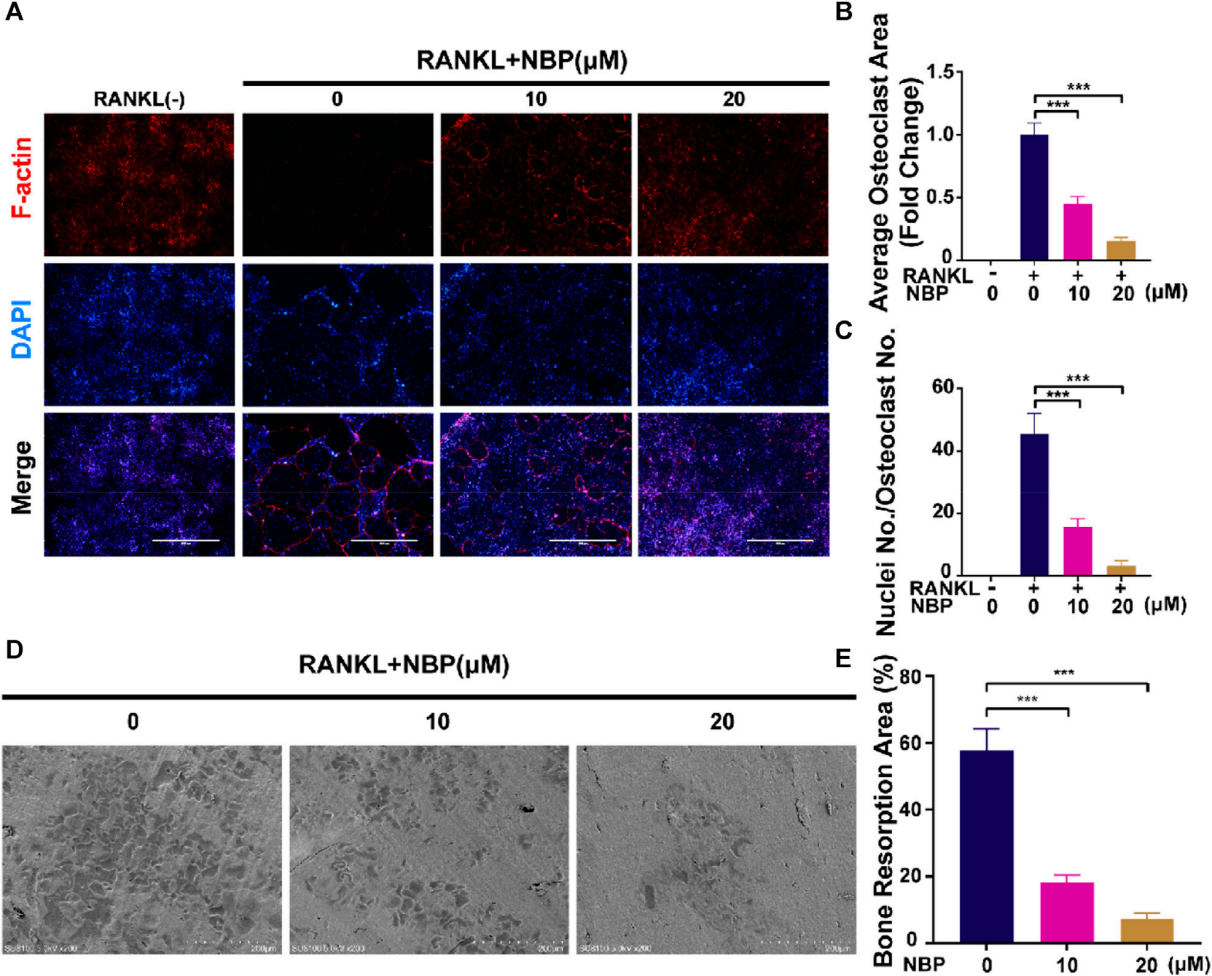
NBP attenuates podosome belt formation and osteoclasts resorption *in vitro*. **(A)** Representative images of NBP inhibition of podosome belt formation (scale bar = 1000 µm). **(B)** Quantification of the average area of osteoclast in different groups of osteoclasts. **(C)** Quantification of the number of nuclei in each group of osteoclasts. **(D)** Representative images of microscopic scans of bone slice (scale bar = 200 µm). **(E)** The resorbed area of bone slices was quantitatively analyzed. *p < 0.05, **p < 0.01, ***p < 0.001 relative to the 0 µM NBP. All data of the bar are presented as the mean ± SD (n = 3 per group).

An illustration of NBP’s molecular structure is found in Figure 1A. To examine the impact of NBP on osteoclasts, we conducted CCK-8 assays to assess the viability of BMMs following 48 and 96 h of treatment. NBP did not impact BMM proliferation at concentrations up to 20 μM (Figures 1B, C). Furthermore, BMMs were RANKL-stimulated and TRAcP-stained after 1 week to evaluate osteoclast differentiation under the effects of various NBP concentrations. We observed a reduction in the formation of multinucleated giant cells with variations in the dosage of NBP (Figures 1D, E). To clarify the main timeframe in which NBP inhibits osteoclastogenesis, 20 μM NBP was administered at different stages of osteoclastogenesis. NBP exerted significant inhibition at all stages, especially during the early and middle phases (Figures 1F, G). Moreover, rhodamine-phalloidin staining revealed effective NBP reduction in the osteoclast area and nuclear number (Figures 2A–C).

### 3.2 NBP effectively reduces bone resorption function

BMMs were cultured on bone slices and exposed to various concentrations of NBP to observe the influence of NBP on the bone resorption function of osteoclasts. The resorption crater areas were then observed by electron microscopy. As the NBP concentration increased, the resorption pit areas on the bone slices gradually decreased (Figures 2D, E). Thus, NBP can effectively reduce osteoclast bone resorption ability *in vitro*.

### 3.3 NBP to prevent OVX-induced bone loss

**FIGURE 3:**
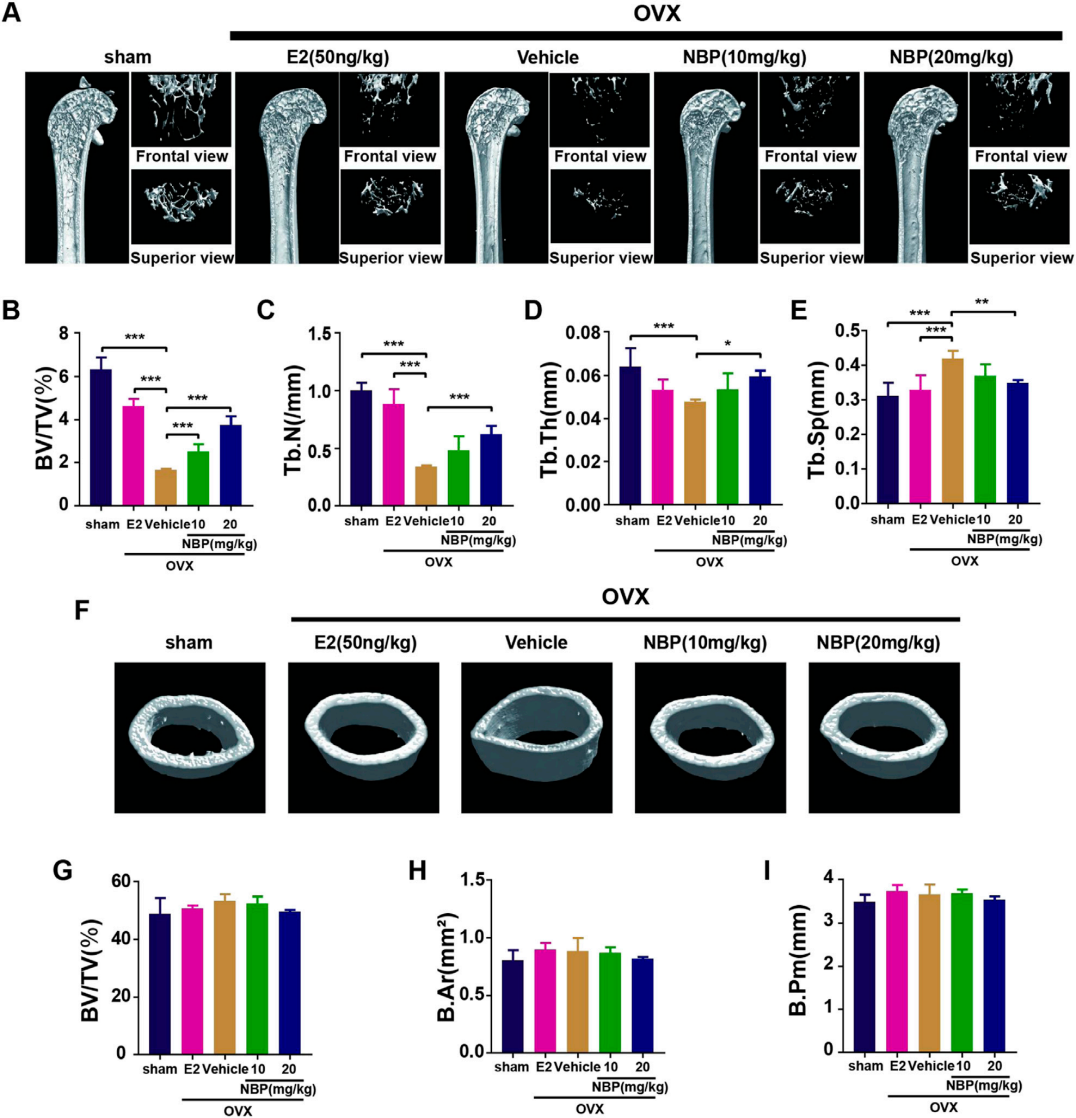
NBP prevents OVX-induced bone loss. **(A)** The representative images of Micro-CT of proximal femur per group. **(B–E)** The parameters related to the bone microstructure (Tb.Th, Tb. Sp, Tb.N, BV/TV) were quantified using Micro-CT software. **(F)** Representative Micro-CT representative images of cortical femur were acquired in various groups. **(G–I)** Quantification of the parameters related to the microstructure of the bone (BV/TV, B.Ar, B.Pm) in the cortical bone of the proximal femur in different groups. *p < 0.05, **p < 0.01, ***p < 0.001 relative to the 0 mg/kg. All data of the bar are presented as the mean ± SD (*n* = 6 per group).

**FIGURE 4:**
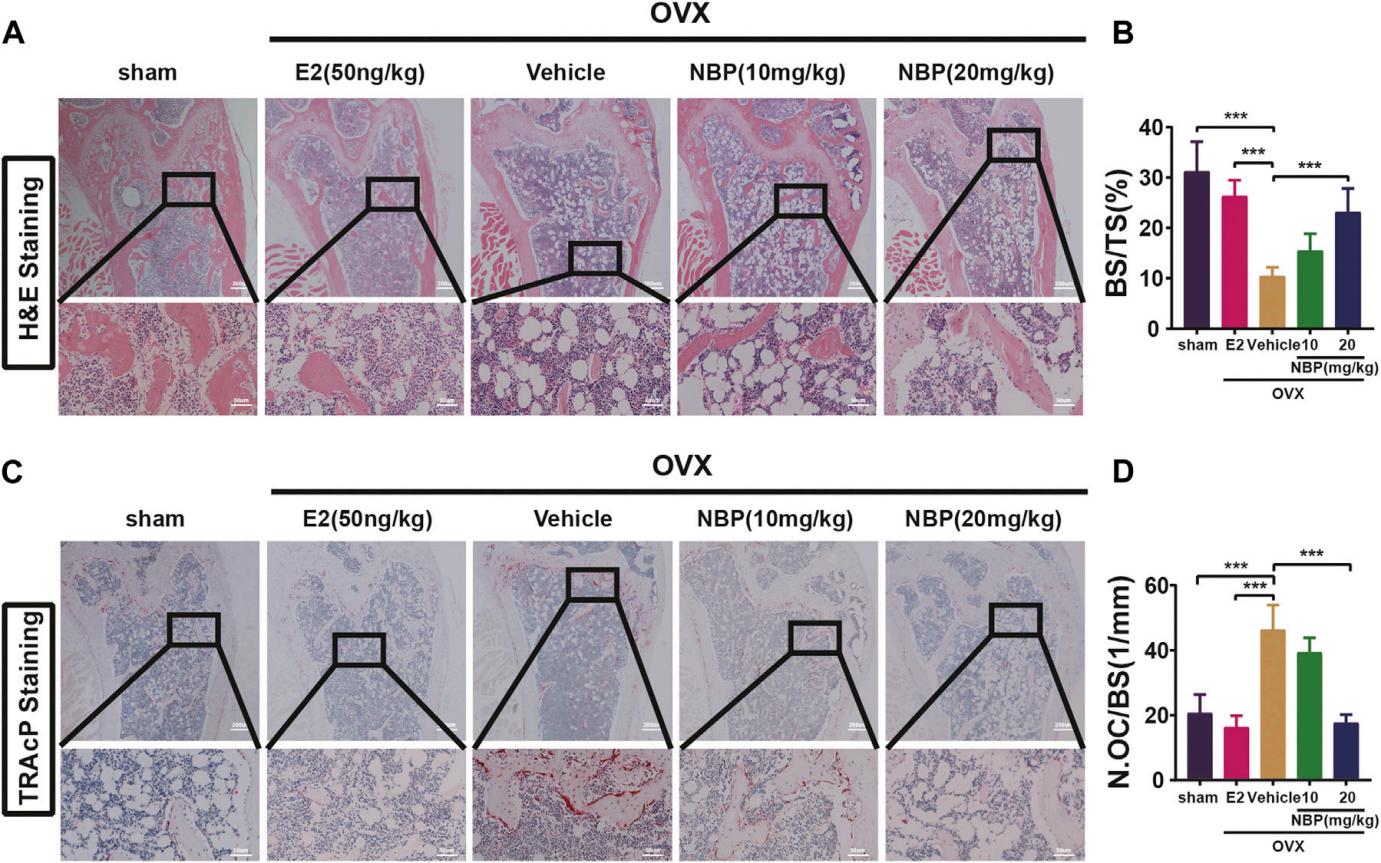
NBP prevents OVX-induced bone loss and reduce osteoclasts. **(A)** Representative HE staning images of femurs in different groups. **(B)** Quantification the percentage of bone trabecular. **(C)** Representative TRAcP staining images of femur. **(D)** Quantification of osteoclast number/bone surface. *p < 0.05, **p < 0.01, ***p < 0.001 relative to the 0 mg/kg. All data of the bar are presented as the mean ± SD (*n* = 3 per group).

This experiment investigated whether NBP could mitigate or reverse osteoporosis *in vivo*. The analysis of femoral microstructural parameters displayed markedly higher values for cancellous bone (Tb.N, BV/TV, Tb.Th) in the sham, E2, and treatment groups than in the untreated OVX group (Figures 3A–E). However, cortical bone parameters (BV/TV, B. Ar, B. Pm) showed no significant intergroup differences (Figures 3F–I). Subsequently, TRAcP staining and H&E staining were performed on specimens to discern the pharmaceutical effects on trabecular and osteoclast quantities within the murine femur. The results showed that NBP significantly prevented bone loss in the OVX model (Figures 4A, B). In addition, TRAcP staining demonstrated that NBP significantly reduced the number of osteoclasts in the femur (Figures 4C, D). The aforementioned results indicated that NBP could reduce estrogen deficiency-induced bone loss.

### 3.4 NBP enhances antioxidant enzyme activity and decreases intracellular ROS levels

**FIGURE 5:**
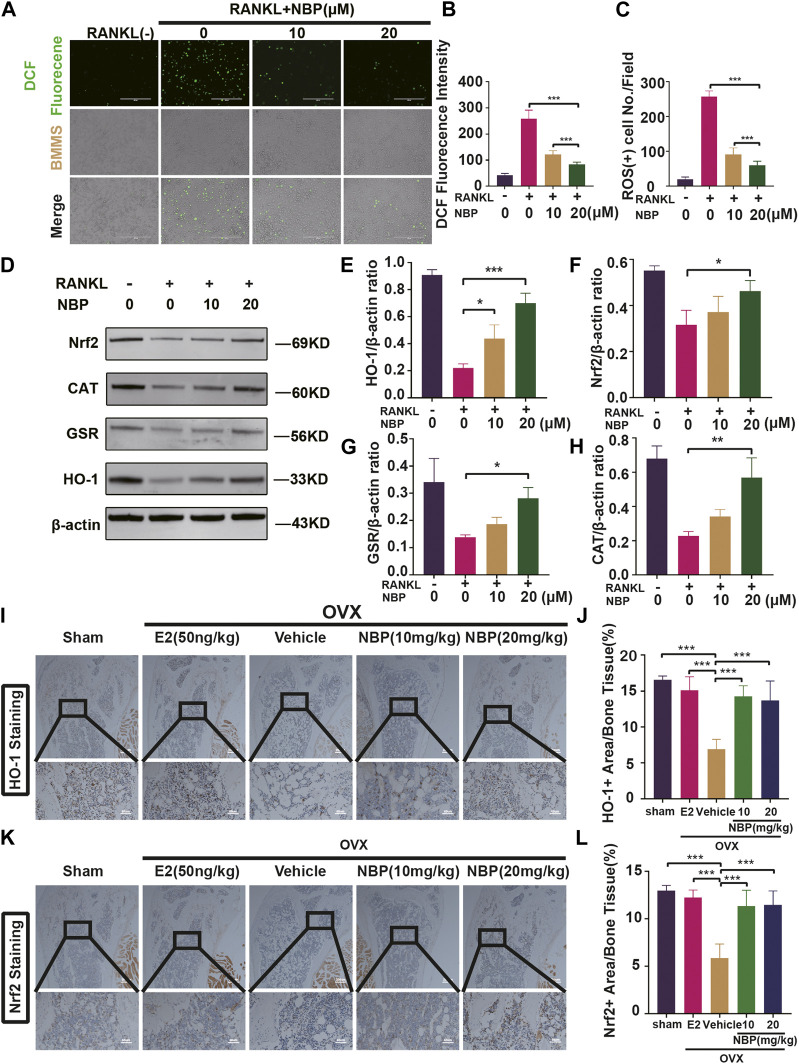
The NBP attenuates ROS in RANKL-stimulated osteoclasts, and elevates the antioxidant enzymes expression both *in vitro* and *in vivo*. **(A)** Representative images of intracellular ROS after RANKL stimulation of osteoclasts. (scale bar = 400 µm) **(B)** Quantification of DCF fluorencence intensity. **(C)** Quantification of the amount of ROS^+^ cells. **(D)** Representative images of Western blot demonstrate the impact of NBP on antioxidant enzymes. **(E–H)** Quantification of the ratios of GSR, Nrf2, HO-1, CAT relative to β-actin. **(I)** Representative images of histologic staining for HO-1 in femoral sections. **(J)** Quantification of the HO-1^+^ area on femoral sections. **(K)** Representative histological staining images for Nrf2 of femurs after treatment with NBP. **(L)** Quantification of the Nrf2^+^ area on femoral sections. *p < 0.05, **p < 0.01, ***p < 0.001 relative to the 0 μM NBP. All data of the bar are presented as the mean ± SD (*n* = 3 per group).

ROS levels were evaluated to investigate the signaling pathways underlying the impact of NBP on osteoclastogenesis. The results showed that NBP effectively reduced intracellular ROS fluorescence intensity and ROS-positive cell numbers 48 h post-RANKL stimulation (Figures 5A–C). As key oxidative stress molecules, Nrf2, HO-1, CAT and GSR were also evaluated. Western blotting revealed that NBP significantly elevated the levels of antioxidant enzymes (Nrf2, CAT, GSR, and HO-1) (Figures 5D–H). Immunohistochemistry of HO-1 and Nrf2 was performed to further explore the antioxidant enzyme effects of NBP on OVX mouse femurs, confirming significant elevation of the antioxidant enzymes *in vivo* (Figures 5I–L). In summary, NBP may reduce ROS and promote the expression of antioxidant enzymes.

### 3.5 NBP downregulates the expression of osteoclast-specific genes and proteins

**FIGURE 6:**
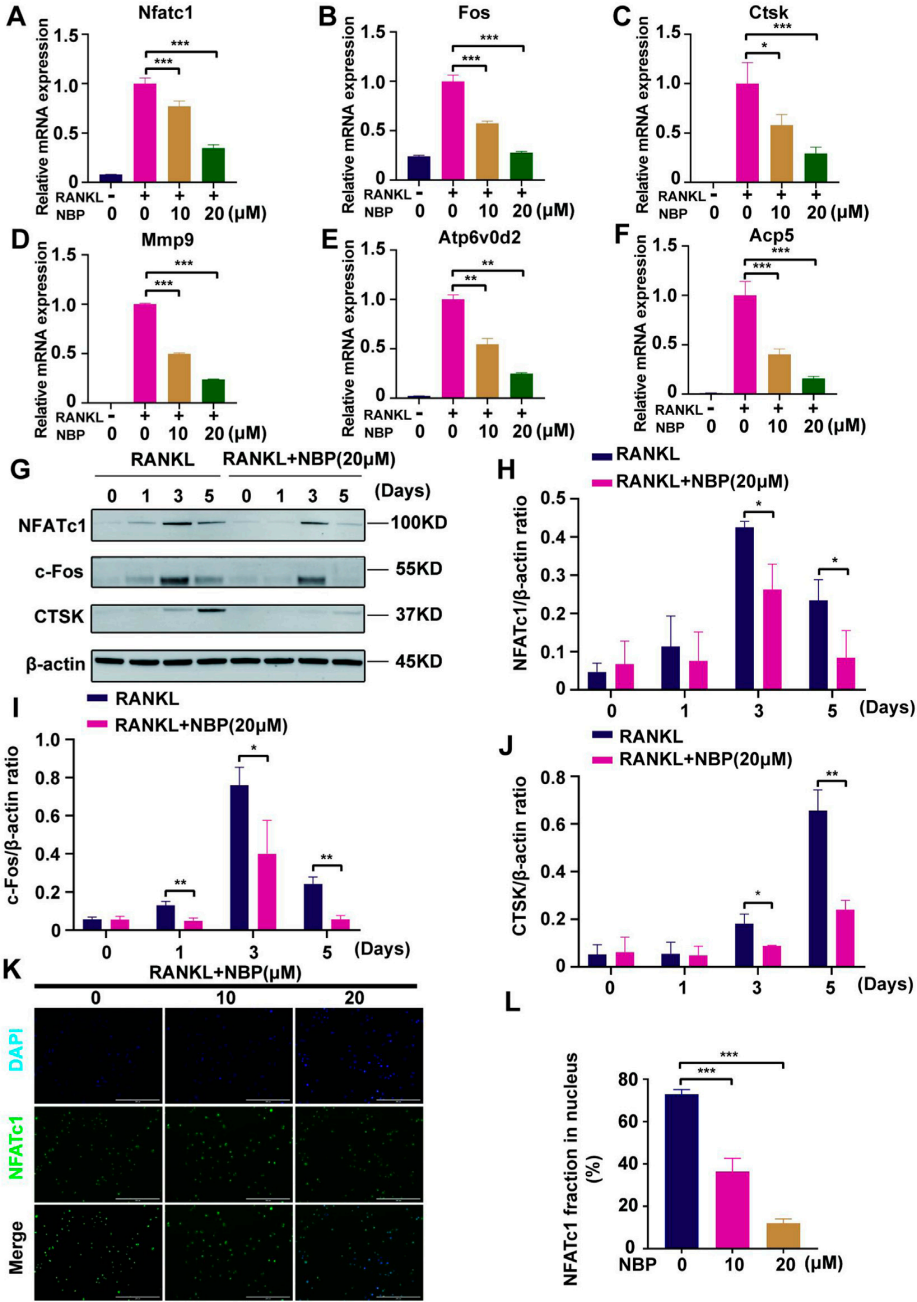
NBP repress the activity and translocation of NFATc1 **(A–F)** Expression of mRNA for Nfatc1, Fos, Ctsk, Mmp9, Atp6v0d2, Acp5 in osteoblasts after RANKL induction. **(G)** Representative images of Western blot demonstrate the impact of NBP on osteoclast-specific proteins (CTSK, c-Fos, NFATc1). BMM were interfered with RANKL in the absence or presence of 20 μM NBP for 0, 1, 3, 5 days. **(H–J)** The expression of the above mentioned proteins was analyzed quantitatively in relation to the β-actin. **(K)** Representative images of NFATc1 nuclear translocation in osteoclasts induced by RANKL (scale bar = 200 μM). **(L)** Quantification of the translocation of NFATc1. *p < 0.05,**p < 0.01,***p < 0.001 relative to the 0 μM NBP. All data of the bar are presented as the mean ± SD (*n* = 3 per group).

Osteoclast-specific gene expression was examined after 5 days of RANKL stimulation. The PCR results demonstrated a significantly decreased expression of osteoclast genes (Nfatc1, Fos, Ctsk, Mmp9, Atp6v0d2, and Acp5) following NBP intervention (Figures 6A–F). Western blotting results showed that NBP significantly downregulated NFATc1 and CTSK on Days 3 and 5 of RANKL stimulation and suppressed c-Fos on Days 1, 3, and 5 (Figures 6G–J). NFATc1 regulates osteoclast genes via nuclear translocation of unphosphorylated NFATc1, and the immunofluorescence results confirmed that NBP effectively inhibited NFATc1 nuclear translocation (Figures 6K, L). Collectively, these results indicate that NBP can potently inhibit the NFATc1 pathway and downregulate osteoclast-specific gene expression.

### 3.6 NBP interferes with the activation of the MAPK pathway induced by RANKL

**FIGURE 7:**
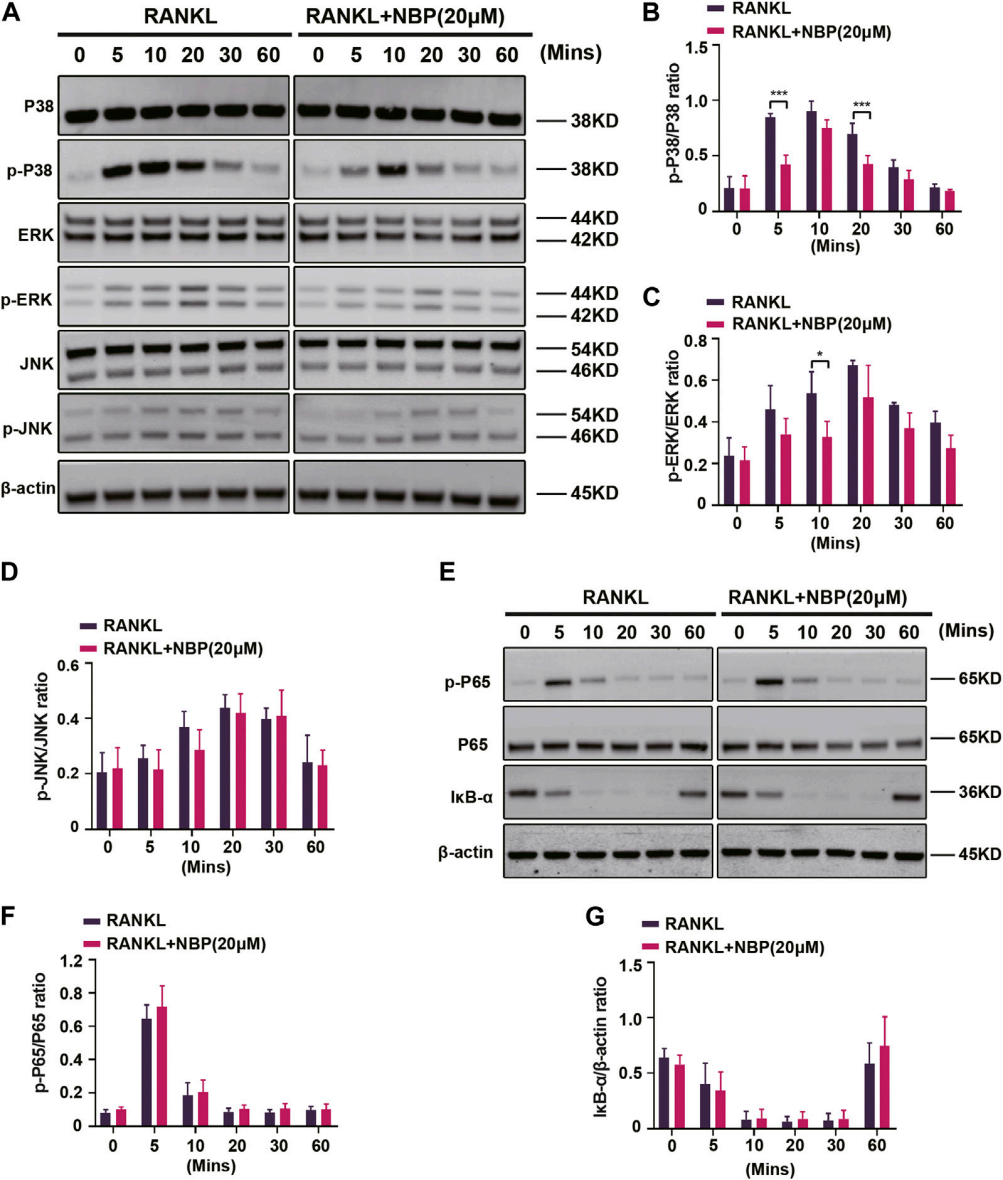
NBP inhibits MAPK signaling in osteoblasts following RANKL induction. **(A)** The expression of MAPK signaling pathway (p-ERK, ERK, p-P38, P38, JNK, p-JNK) and β-actin following NBP intervention were evaluated by Western blot. **(B–D)** The expression of the above mentioned proteins was analyzed quantitatively in relation to the β-actin. **(E)** The expression of NF-κB signaling pathway (IκB-α, P65, and p-P65) and β-actin following NBP intervention were evaluated by Western blot. **(F,G)** The expression of the NF-κB signaling pathway was analyzed quantitatively in relation to the β-actin. *p < 0.05, **p < 0.01, ***p < 0.001 relative to the 0 μM NBP. All data of the bar are presented as the mean ± SD (*n* = 3 per group).

The key osteoclastogenic MAPK and NF-κB pathways were evaluated. Western blotting revealed that NBP effectively inhibited p38 activation and phosphorylation at 5 and 20 min of RANKL stimulation and ERK phosphorylation at 10 min, while JNK phosphorylation showed no significant change (Figures 7A–D). Conversely, NF-κB pathway P65 phosphorylation and IκB-α expression were unaltered (Figures 7E–G). The results suggest that NBP inhibits osteoclastogenesis partially through the MAPK pathway rather than the NF-κB pathway.

### 3.7 NBP is not involved in forming and mineralizing bone

**FIGURE 8:**
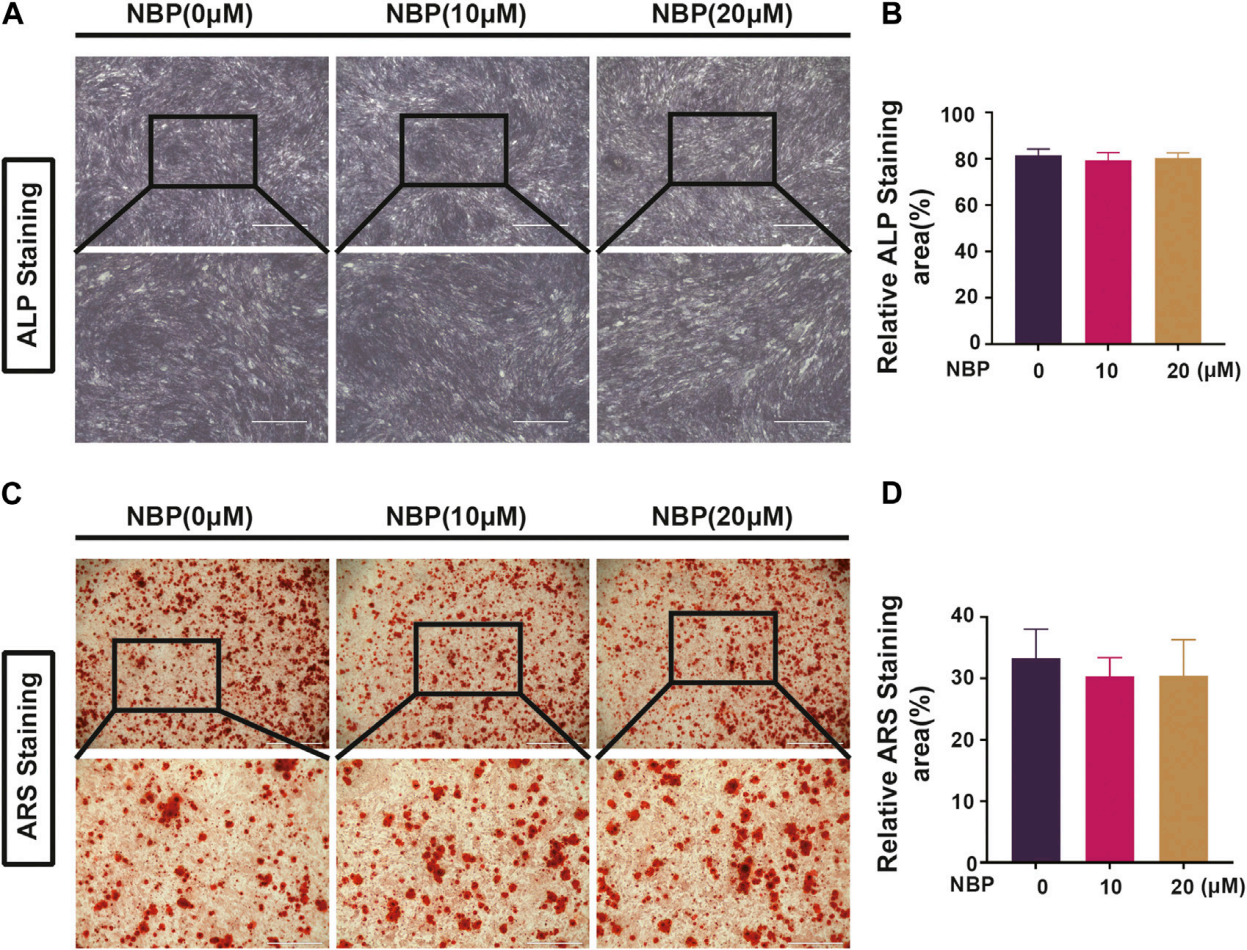
NBP is not involved in forming and mineralizing bone. **(A)** Representative ALP staining images of MC3T3-E1 cells after intervention with different concentrations of NBP (scale bar = 1000 μM). **(B)** Quantification of ALP in osteoblasts after NBP intervention. **(C)** Representative ARS staining images of MC3T3-E1 cells after intervention with different concentrations of NBP (scale bar = 1000 μM). **(D)** Quantification of ARS in osteoblasts after NBP intervention. *p < 0.05, **p < 0.01, ***p < 0.001 relative to the 0 μM NBP. All data of the bar are presented as the mean ± SD (*n* = 3 per group).

Osteoclast and osteoblast activity conjointly regulate bone mass homeostasis, and the effects of NBP on osteoblasts were evaluated. MC3TC cells were cultured with NBP for 7 days and then stained with ALP, revealing that osteoblast differentiation was unaffected by NBP (Figures 8A, B). ARS staining was conducted 21 days following cultivation to evaluate mineralization status, which also demonstrated no significant intergroup differences in osteoblast mineralization (Figures 8C, D). Therefore, NBP did not have a significant impact on the differentiation or mineralization of osteoblasts.

## 4 Discussion

Osteoporosis is a metabolic bone disease distinguished by reduced bone density and deterioration of bone structure. It affects the entire body and can result in fractures and increased morbidity risk (Yao et al., 2021). Considering the global aging population, this condition represents a growing health burden (Ryoo et al., 2020; Xu et al., 2021). Furthermore, osteoclast activity, leading to bone loss, is a significant contributor to the pathogenesis of osteoporosis (Jacome-Galarza et al., 2019; Shih et al., 2019; Palui et al., 2022). Hence, osteoclasts remain the main target in osteoporosis treatment. In recent years, osteoporosis treatment has mainly comprised antiresorptive drugs (bisphosphonates, strontium salts) and bone anabolic compounds [parathyroid hormone peptides PTH (1-84 or 1-34)] (Kroupova et al., 2023). However, prolonged use of these agents may cause side effects leading to drug discontinuation, which highlights the need for a safe, efficacious alternative (Zhang et al., 2023). Recently, numerous herbal extracts have been researched as potential anti-osteoporosis therapeutic options owing to their potent effects and few side effects (Takagi et al., 2022; Zheng et al., 2022; Xu et al., 2023). This study presents novel findings showing that NBP suppresses osteoclast differentiation and bone resorption *in vitro* through reducing RANKL-induced ROS levels and blocking the MAPK signaling pathway. Furthermore, it mitigated or prevented OVX-induced bone loss *in vivo*.

To investigate the impact of NBP on osteoclasts *in vitro*, we conducted experiments on osteoclastogenesis and bone resorption. Following NBP intervention *in vitro*, we observed that NBP effectively suppressed osteoclastogenesis and bone resorption. On the basis of the results of the *in vitro* studies, we established an OVX model to simulate the physiological features of postmenopausal women. An animal model was utilized to evaluate the anti-osteoporotic efficacy of NBP *in vivo*. The Micro-CT and HE results demonstrated a significant improvement in parameters of bone trabecular (Tb. Th, BV/TV, Tb. N) in the treatment group. Additionally, the results of TRAcP staining indicated that NBP reduced the number of TRAcP+ osteoclasts *in vivo*. The above findings showed that NBP demonstrated a substantial protective impact against bone loss induced by OVX. However, it is worth further investigating whether NBP is efficient for other types of osteoporosis.

RANKL receptor binding initiates signaling cascades, recruiting TNF receptor-associated Factor 6 (TRAF6) to mediate transcription factor kappa B (NF-κB), c-Jun, and P38/c-fos expression, thereby activating the NFATc1 transcription factor (Kim et al., 2020; Russo et al., 2020; Kitazawa et al., 2021). NFATc1 is an essential osteoclastogenic NFAT family member that undergoes N-terminal dephosphorylation by calcineurin, inducing nuclear translocation (Chen X. et al., 2019; Tong et al., 2022). Its C-terminus then binds to specific DNA sequences alongside activator protein 1 (Mcdonald et al., 2021; Yao et al., 2021). Once activated, NFATc1 translocates from the cytoplasm into the nucleus to transcribe osteoclast genes (Xiang et al., 2019; Colombo et al., 2022; Nannan et al., 2023). Thus, NFATc1 is pivotal for osteoclastogenesis. We conducted experiments on the induction of protein expression and nuclear translocation of the NFATc1 pathway in osteoclasts stimulated by RANKL. The results revealed that NBP significantly inhibited osteoclast genes (Fos, Ctsk, Mmp9, Atp6v0d2, and Acp5) by suppressing NFATc1 pathway proteins and nuclear translocation, thereby downregulating osteoclastogenesis.

Upon RANKL recognition by its receptor RANK, TRAF6 binds to the cytoplasmic domain, activating NF-κB inhibitor kinases (IKKs) to phosphorylate IκB serine motifs and activate NF-κB (Li X. et al., 2021; Jimi and Katagiri, 2022). Nuclear NF-κB enables P50/P65 dimerization and upregulates c-fos and NFATc1, inducing osteoclast gene transcription. MAPKs consist of the serine/threonine protein Jun N-terminal kinase (JNK), p38 and kinases extracellular signal-regulated kinase (ERK1/2) (Zhang et al., 2022b; Dong et al., 2022). The MAPK pathway impacts gene expression, mitosis, differentiation, and apoptosis (Devi and Shanmugarajan, 2023). Like NF-κB, TRAF6 activation by RANK/RANKL binding stimulates ASK1 to phosphorylate the MAPK cascade, regulating signaling and the three key pathways (Wong et al., 2020). Nuclear translocation-activated ERK is particularly important for osteoclastogenesis, while P38 and JNK phosphorylation respond to RANKL by regulating AP-1 and phosphorylating c-Jun and c-fos transcription (Zhang et al., 2018; Wang et al., 2023; Yu et al., 2023). In this study, both P38 and ERK phosphorylation were inhibited by NBP but did not affect JNK or NF-κB activation. Therefore, our findings suggest that NBP impedes osteoclastogenesis and function through the MAPK pathway instead of the NF-κB pathway.

Reactive oxygen species (ROS), known as important intracellular second messengers, play significant roles not only in cellular processes such as cell differentiation and inflammation but also as key transcription factors in osteoclast differentiation. RANKL stimulation of osteoclast precursors activates TRAF6 and Nox1, upregulating Keap1 to decrease the Nrf2/Keap1 ratio (Srivastava and Sapra, 2022). Meanwhile, decreased Nrf2/Keap1 further downregulates antioxidant enzyme expression and increases the levels of ROS (Kanzaki et al., 2016). Growing evidence suggests that ROS induced by RANKL regulate the MAPK, PI3K, and NFKB pathways in osteoclasts, thereby promoting osteoclastogenesis and differentiation. Consequently, the effective removal of intracellular ROS becomes crucial for determining the fate of osteoclasts. In the normal state, the DGR region of keap1 and the Neh2 region of Nrf combine to maintain stable anchoring of Nrf2 to the cytoskeleton. When the levels of intracellular ROS increase, Nrf2 dissociates from keap1 and translocates across the nuclear membrane to identify the antioxidant response element (ARE), thereby enhancing the expression of crucial antioxidant enzymes such as HO-1, GSR, and CAT. These enzymes are pivotal in the process of ROS scavenging and inflammation regulation. Among them, HO-1 is a key antioxidant inhibiting RANKL-induced osteoclastogenesis, while GSR is a potent scavenger of intracellular ROS (Leon-Reyes et al., 2023). CAT is responsible for breaking down hydrogen peroxide to reduce ROS (Galasso et al., 2021). Our study demonstrated that NBP can decrease excess osteoclastogenic ROS and increase the expression of HO-1, GSR, and CAT antioxidant enzymes, as confirmed by immunohistochemical staining. These results suggest that NBP may repress osteoclast differentiation by scavenging ROS and promoting the expression of antioxidant enzymes.

As mentioned above, NBP is closely related to both the MAPK pathway and ROS in osteoclasts. It has been shown that reactive oxygen species (ROS) is a key messenger that not only directly activates the MAPK pathway *in vivo*, but also indirectly affects the MAPK pathway through interactions with other signaling molecules such as RANKL and NF-κB. Therefore, the inhibition of osteoclastogenesis by NBP is directly related to the reduction of ROS levels and the inhibition of MAPK pathway after RANKL stimulation.

As previously discussed, osteoblasts are also a key determinant of the state of bone homeostasis. This result for osteoblasts revealed that NBP did not significantly affect osteoblast differentiation or mineralization.

**FIGURE 9:**
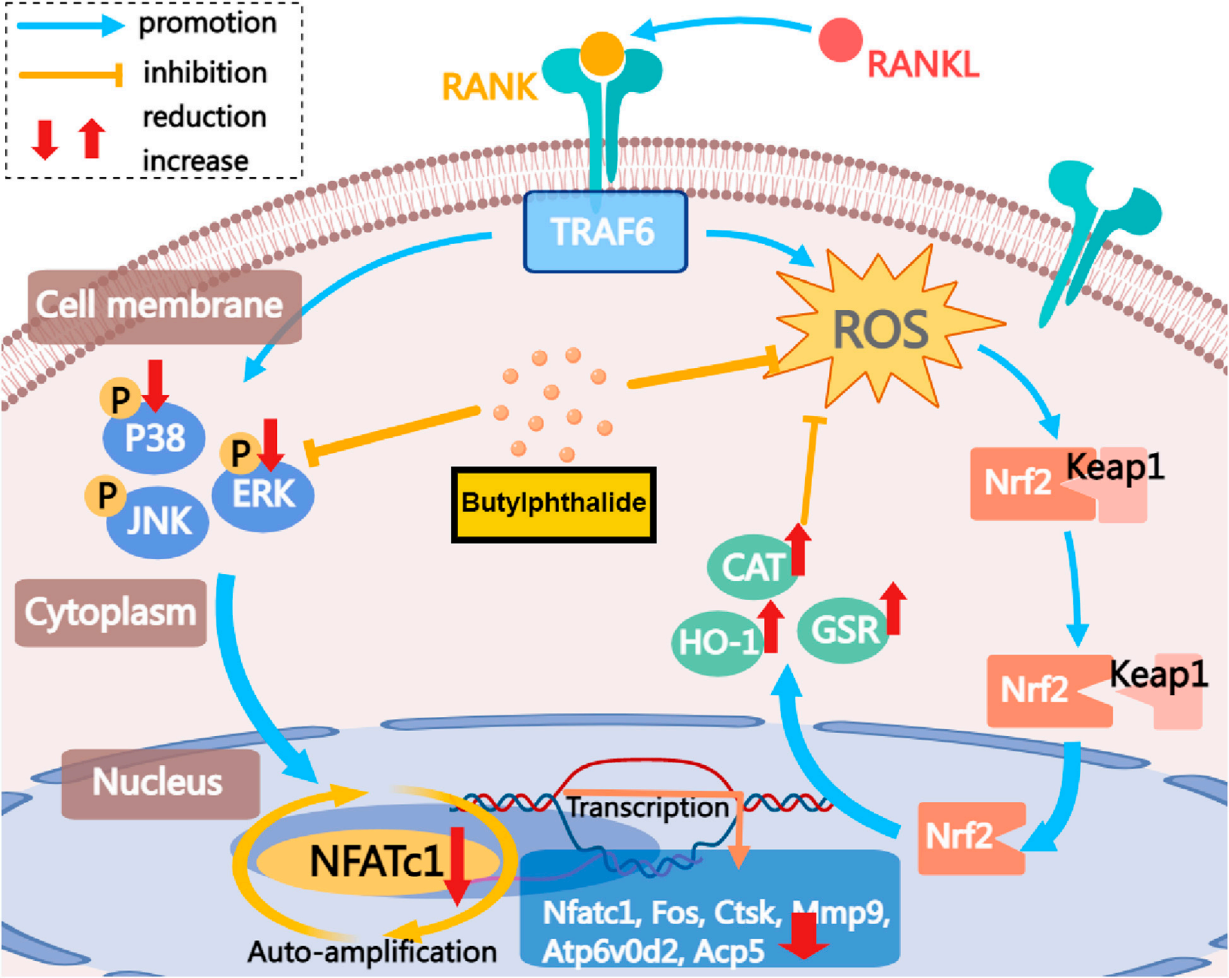
The mechanism by which NBP inhibits osteoclastogenesis is demonstrated. This study demonstrates that NBP inhibits MAPK pathway phosphorylation, reduces ROS, increases antioxidant enzyme levels, and ultimately decreases osteoclast formation and bone resorption by down-regulating the NFATc1 signaling pathway.

In summary, this study indicates that NBP inhibits NFATc1 and MAPK signaling pathways by reducing ROS in osteoclasts (Figure 9). This inhibition is achieved by elevating the expression and activity of antioxidant enzymes. Consequently, it suppresses the expression of crucial genes and proteins in osteoclasts, leading to the suppression of osteoclast formation and function. NBP also prevented OVX-induced estrogen-deficient bone loss *in vivo*. In conclusion, these findings could contribute to the potential development of NBP-targeted therapeutic treatments for osteoporosis and other skeletal diseases.

## Data Availability

The original contributions presented in the study are included in the article/Supplementary Material, further inquiries can be directed to the corresponding authors.
