# Supplementary figures and images for: Unveiling the potential of Butylphthalide: inhibiting osteoclastogenesis and preventing bone loss
Source: Front Pharmacol. 2024 Feb 23;15:1347241. doi: 10.3389/fphar.2024.1347241 (PMC10922197; doi:10.3389/fphar.2024.1347241)

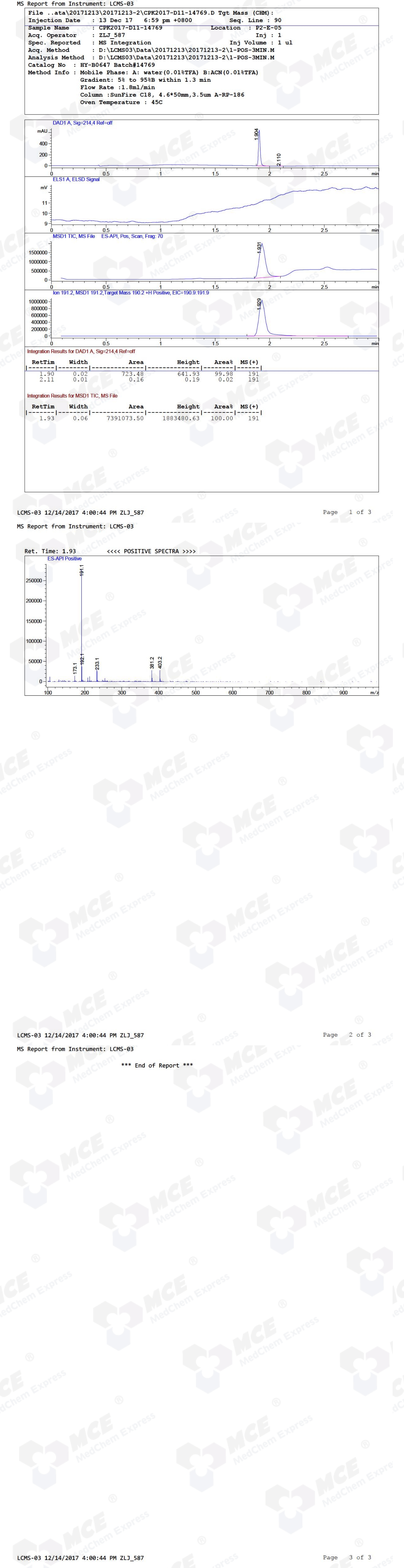

Supplement: Supplementary file 1 [file Image3.JPEG]

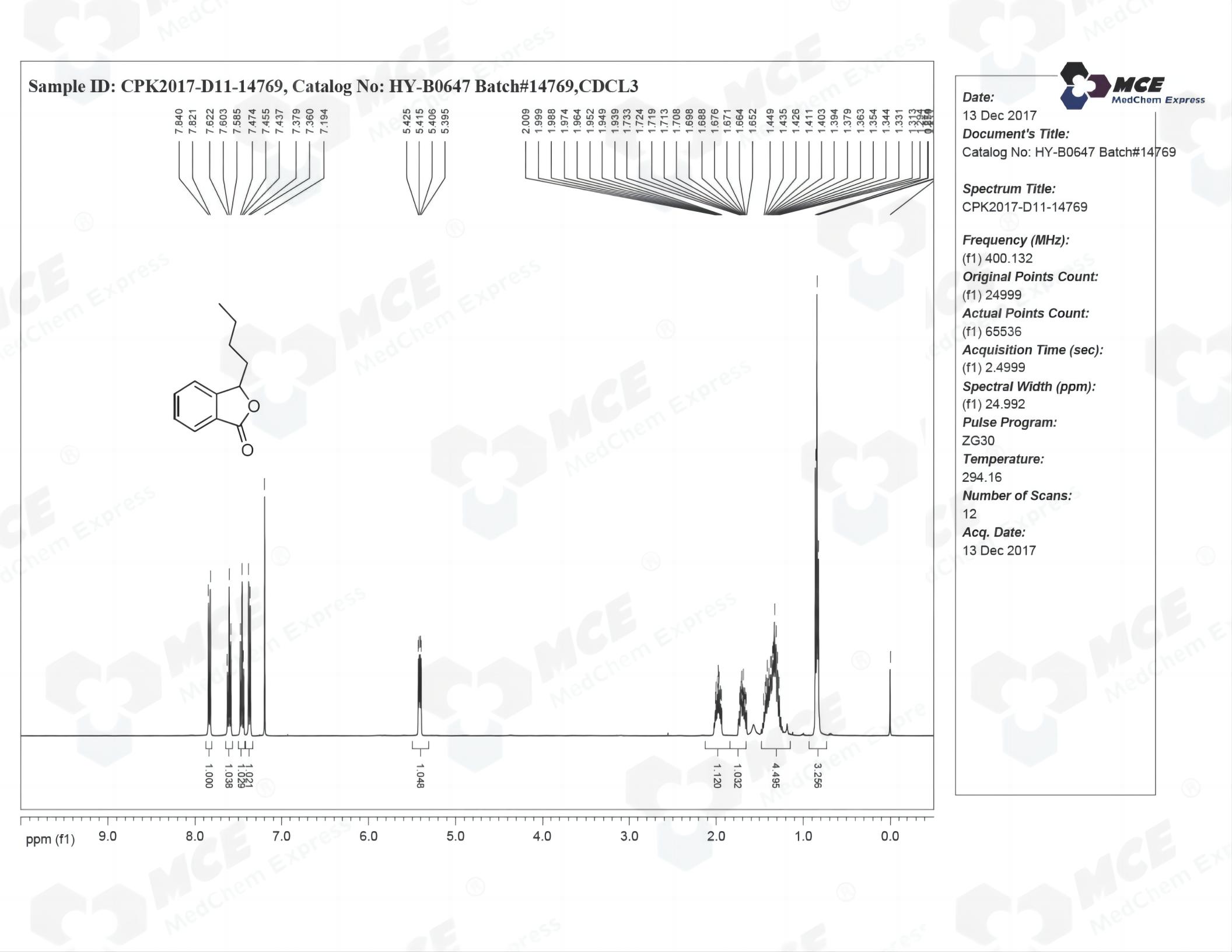

Supplement: Supplementary file 2 [file Image2.JPEG]

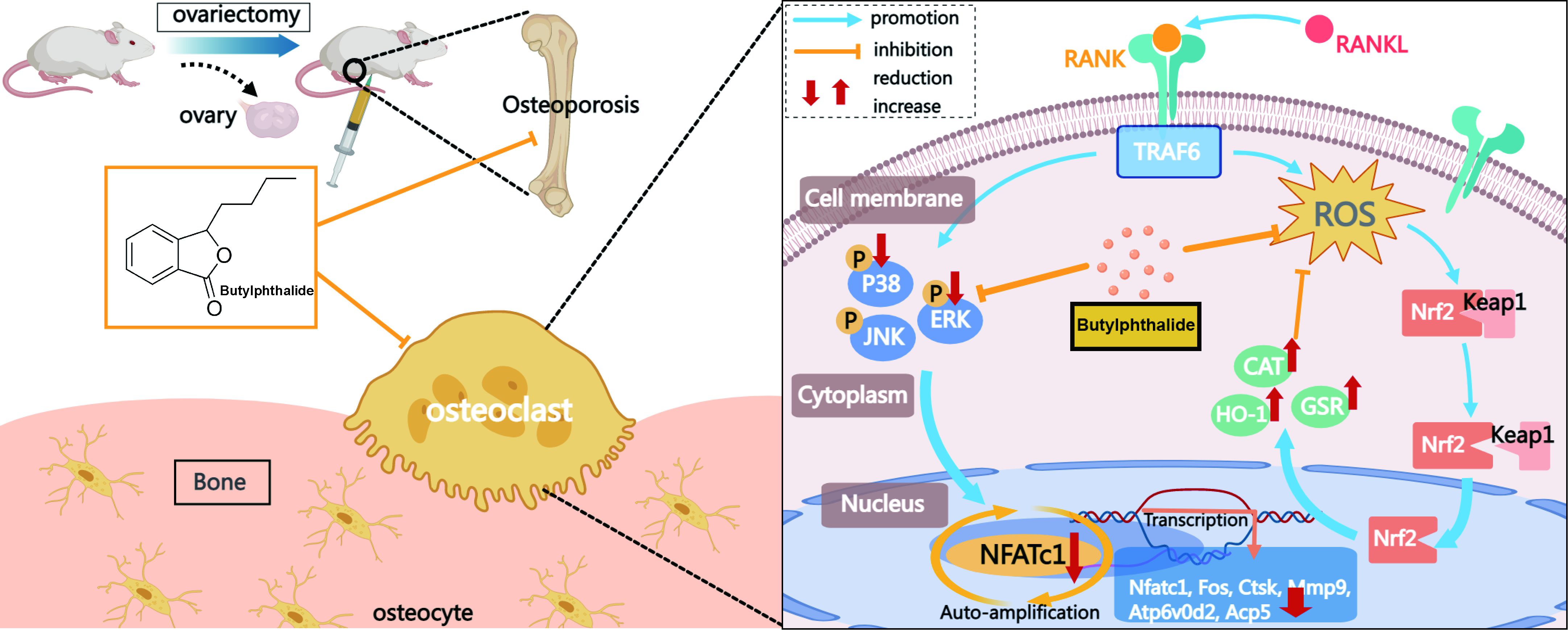

Supplement: Supplementary file 3 [file Image1.TIF]
