# Supplementary material for: Unveiling the potential of Butylphthalide: inhibiting osteoclastogenesis and preventing bone loss
Source: Front Pharmacol. 2024 Feb 23;15:1347241. doi: 10.3389/fphar.2024.1347241 (PMC10922197; doi:10.3389/fphar.2024.1347241)
Supplement: Supplementary file 4 [file Table1.DOC]

| **Gene** | **NCBI ID** | **Primer Sequence(5-3’)** |  |
| --- | --- | --- | --- |
|  |  | **Forward** | **Reverse** |
| Nfatc1 | 18018 | GGTGCTGTCTGGCCATAACT | GAAACGCTGGTACTGGCTTC |
| Fos | 14281 | CCAGTCAAGAGCATCAGCAA | AAGTAGTGCAGCCCGGAGTA |
| Ctsk | 13038 | AGGCGGCTCTATATGACCACTG | TCTTCAGGGCTTTCTCGTTC |
| Mmp9 | 17395 | CGTGTCTGGAGATTCGACTTGA | TTGGAAACTCACACGCCAGA |
| Atp6v0d2 | 242341 | GTCCCATTCTTGAGTTTGAGG | GGATAGAGTTTGCCGAAGGTT |
| Acp5 | 11433 | TGTGGCCATCTTTATGCT | GTCATTTCTTTGGGGCTT |
| GAPDH | 14433 | GGCACAGTCAAGGCTGAGAATG | ATGGTGGTGAAGACGCCAGTA |

**Table 1 The specific primers used in qRT-PCR**
